# Identification of Altered Plasma Proteins by Proteomic Study in Valvular Heart Diseases and the Potential Clinical Significance

**DOI:** 10.1371/journal.pone.0072111

**Published:** 2013-08-27

**Authors:** Ge Gao, Chao Xuan, Qin Yang, Xiao-Cheng Liu, Zhi-Gang Liu, Guo-Wei He

**Affiliations:** 1 TEDA International Cardiovascular Hospital and The Affiliated Hospital, Hangzhou Normal University, Tianjin and Hangzhou, China; 2 TEDA International Cardiovascular Hospital, Tianjin, China; 3 Department of Medicine and Therapeutics, The Chinese University of Hong Kong, Hong Kong, China; 4 Department of Surgery, Oregon Health and Science University, Portland, Oregon, United States of America; The Chinese University of Hong Kong, Hong Kong

## Abstract

**Background:**

Little is known about genetic basis and proteomics in valvular heart disease (VHD) including rheumatic (RVD) and degenerative (DVD) valvular disease. The present proteomic study examined the hypothesis that certain proteins may be associated with the pathological changes in the plasma of VHD patients.

**Methods and Results:**

Differential protein analysis in the plasma identified 18 differentially expressed protein spots and 14 corresponding proteins or polypeptides by two-dimensional electrophoresis and mass spectrometry in 120 subjects. Two up-regulated (complement C4A and carbonic anhydrase 1) and three down-regulated proteins (serotransferrin, alpha-1-antichymotrypsin, and vitronectin) were validated by ELISA in enlarging samples. The plasma levels (n = 40 for each) of complement C4A in RVD (715.8±35.6 vs. 594.7±28.2 ng/ml, *P* = 0.009) and carbonic anhydrase 1 (237.70±15.7 vs. 184.7±10.8 U/L, *P* = 0.007) in DVD patients were significantly higher and that of serotransferrin (2.36±0.20 vs. 2.93±0.16 mg/ml, *P* = 0.025) and alpha-1-antichymotrypsin (370.0±13.7 vs. 413.0±11.6 µg/ml, *P* = 0.019) in RVD patients were significantly lower than those in controls. The plasma vitronectin level in both RVD (281.3±11.0 vs. 323.2±10.0 µg/ml, *P* = 0.006) and DVD (283.6±11.4 vs. 323.2±10.0 µg/ml, *P* = 0.011) was significantly lower than those in normal controls.

**Conclusions:**

We have for the first time identified alterations of 14 differential proteins or polypeptides in the plasma of patients with various VHD. The elevation of plasma complement C4A in RVD and carbonic anhydrase 1 in DVD and the decrease of serotransferrin and alpha-1-antichymotrypsin in RVD patients may be useful biomarkers for these valvular diseases. The decreased plasma level of vitronectin – a protein related to the formation of valvular structure – in both RVD and DVD patients might indicate the possible genetic deficiency in these patients.

## Introduction

Valvular heart disease (VHD) is the subject of growing attention in the field of cardiovascular medicine, particularly because of the changes that have occurred in its presentation and management over the past 60 years. Since the 1950s, the predominance of valvular disease has shifted from rheumatic valvular disease (RVD) to degenerative valvular disease (DVD) in industrialized countries, leading to important changes in patient characteristics and in the distribution of the type of valvular lesions [1]–[3]. However, even recently, VHD encompassing a number of common cardiovascular conditions still accounts for 10% to 20% of all cardiac surgical procedures in the United States [4]. In contrast, in developing countries VHD is still mainly caused by rheumatic heart disease [5], although the percentage of heart valve surgery for DVD in these countries is also increasing. In fact, in China, the incidence of rheumatic heart disease is reported to be as high as 0.2% of the adult population, 10 times higher than that in developed countries [6], and China is estimated to have about 2 million adult rheumatic heart disease patients [6].

The genetic basis and proteomics of common VHD are important to provide information for diagnosis and treatment of these diseases. Proteomics is a powerful tool to describe changes in protein expression developing a map of proteins, and more importantly, proteomics allows us to identify protein isoforms in plasma. In fact, plasma is the ideal source for proteome analysis as it is easily sampled from patients and reflects processes in anatomical compartments. As a complex body fluid, more than 10,000 different proteins are present in human plasma and many of them are secreted or shed by cells during different physiological or pathological processes [7]. Plasma is expected to be an excellent source of protein biomarkers because it circulates through, or comes in contact with all tissues. During this contact it is likely to pick up proteins secreted or shed by tissues [8].

The proteome reflects all proteins and peptides that may be related to certain genes and allows a more detailed evaluation of disease status. The fundamental problem in proteomics is the individuality of different proteins. Separation of large numbers of proteins is normally done by two-dimensional electrophoresis (2-DE). The resulting proteins which are separated with 2-DE are normally identified by mass spectrometry, especially the matrix-assisted laser desorption-ionization time-of-flight mass spectrometry (MALDI/TOF MS) [9], as we recently reported in our work on congenital heart diseases [10].

However, although there were few proteomic studies on VHD [11]–[15], most of them studied tissues and there were only handful studies on RVD [14]. Further, there were no reports on the proteomic study on the comparison between DVD and RVD. Thus, in the present study, 2-DE and MALDI/TOF MS were for the first time employed to investigate protein expression alterations in both RVD and DVD with comparison to control plasma samples from normal subjects in order to identify the pathological changes. From these analyses, it is possible to derive the surrogate biomarkers of disease processes that may ultimately affect patient outcomes.

## Materials and Methods

### Ethics Statement

The protocol was approved by the Ethics Committee of TEDA International Cardiovascular Hospital and written informed consent was obtained from all of the individuals participated in this study. This consent procedure was approved by the Ethics Committee of TEDA International Cardiovascular Hospital.

### Clinical Features and Population Study

From January 2011 to April 2012, 120 subjects including 40 RVD patients, 40 DVD patients and 40 with same ethic, gender, age and no reported cardiac phenotype controls were recruited in the study at TEDA-International Cardiovascular Hospital, Tianjin, China. The protocol was approved by the Ethics Committee of the hospital and informed consent was obtained from all of these subjects. The demographics of the VHD patients and controls are shown in Table 1. Clinical assessment of the patients was performed, including anthropometric measurement, physical examination, echocardiographic, and radiological evaluation. All patients underwent corrective surgery at TEDA-International Cardiovascular Hospital, Tianjin, China and the diagnosis was confirmed during surgery. There were no differences in demographics of the study population; however, the aortic diameter was larger in the DVD than in RVD patients (34.6±1.5 vs. 29.5±1.1, p<0.05, Table 2). Similarly, the left ventricular-diastolic diameter was larger in the DVD than in the RVD patients (60.7±1.7 vs. 49.7±2.2, p<0.05, Table 2). This probably reflects the pathological differences between RVD and DVD because more patients in DVD group had severe aortic stenosis or incompetence that usually cause more severe left ventricular hypertrophy. Table 3 shows the diversity of the percentage of the operations performed for those patients. The DVD patients had higher percentage for aortic valve replacement that is obviously correlated to the higher aortic diameter and hypertrophic left ventricle indicated by larger left ventricular-diastolic diameter, as mentioned above.

**Table 1:** Demographics of the Study Population.

	Control	RVD	DVD
**Age, years**	52.1±1.6	51.8±1.5	53.3±1.4
**Sex, M/F**	20/20	20/20	20/20
**GLU, mmol/L**	5.63±0.24	5.06±0.24	5.32±0.49
**TBIL, µmol/L**	12.02±0.86	17.57±2.95	13.79±0.83
**GPT, U/L**	19.50±3.29	24.70±3.98	17.75±1.97
**TCHOL, mmol/L**	5.51±0.26	5.03±0.20	5.01±0.21
**TG, mmol/L**	1.51±0.14	1.19±0.16	1.24±0.23
**HDL-C, mmol/L**	1.28±0.06	1.25±0.06	1.29±0.08
**LDL-C, mmol/L**	3.73±0.22	3.37±0.18	3.25±0.18
**UA, µmol/L**	335.6±18.7	348.9±20.9	353.8±25.9
**UREA, mmol/L**	4.91±0.31	5.19±0.32	5.52±0.32
**CREA, µmol/L**	63.35±2.96	63.90±2.69	64.85±2.45

All variables displayed as mean ± SEM (n = 40 in each group);

RVD = rheumatic valvular disease;

DVD = degenerative valvular disease; M = male; F = female; GLU = glucose; TBIL = total bilirubin;

GPT = glutamic-pyruvic transaminase; TCHOL = total cholesterol; TG = triglyceride; HDL-C = high-density lipoprotein cholesterol; LDL-C = low-density lipoprotein cholesterol; UA = uric acid; CREA = creatinine.

P>0.05 in all comparisons between any two of these groups (unpaired t-test for all continuous numbers).

**Table 2:** Echocardiographic Findings of the Patients.

	RVD	DVD
**AAO-D, mm**	29.5±1.1	34.6±1.5*
**IVS-Td, mm**	10.3±0.4	11.7±0.6
**LVPW-Td, mm**	10.0±0.4	11.0±0.5
**LV-Dd, mm**	49.7±2.2	60.7±1.7*
**LA-Ds_(A-P)_, mm**	50.3±2.3	46.2±1.3
**MPA-Ds, mm**	27.5±0.7	27.7±0.8
**RV-Dd_(L-R)_, mm**	33.7±1.1	34.6±0.8
**RA-Ds_(L-R)_, mm**	38.3±2.0	36.6±1.1

RVD = rheumatic valvular disease;

DVD = degenerative valvular disease;

AAO-D: diameter of the ascending aorta;

IVS-Td: interventricular septum- thickness at end-diastole;

LVPW-Td: left ventricular posterior wall- thickness at end-diastole;

LV-Dd: left ventricular-diastolic diameter;

LA-Ds(A-P): left atrium-systolic diameter (anterior-posterior);

MPA-Ds: main pulmonary artery- systolic diameter;

RV-Dd(L-R): right ventricular-diastolic diameter (left-right);

RA-Ds(L-R): right atrium-systolic diameter (left-right).

* P<0.05.

**Table 3:** Operation Performed in Each Group of Patients.

	RVD		DVD	
	n	%	n	%
**MVR (±TVR)**	22 (8)	55 (20)	20 (4)	50 (10)
**AVR (±TVR)**	0 (0)	0 (0)	16 (0)	40 (0)
**MVR+AVR (±TVR)**	18 (4)	45 (10)	4 (0)	10 (0)

RVD = rheumatic valvular disease;

DVD = degenerative valvular disease;

MVR: mitral valve replacement;

**±**TVR: with or without concomitant tricuspid valve repair;

AVR: aortic valve replacement.

### Plasma Samples

Plasma samples were collected from 120 subjects including 40 RVD patients, 40 DVD patients, and 40 controls. From each plasma sample, 2 ml blood was allowed to clot at 4°C for at least 2 h and then centrifuged at 1500 g for 10 min to reach sediment the clotted cells. Plasma was then collected, divided into aliquots, and stored frozen at −80°C until the analysis was carried out.

### Plasma High-Abundance Protein Depletion

Pooling plasma samples from 20 RVD patients, 20 DVD patients, and 20 healthy controls were processed to deplete the top-two (albumin, IgG) high abundance proteins using the ProteoExtract™ Albumin/IgG Removal Kit (Calbiochem, La Jolla, CA, USA). Samples were processed according to the manufacturer's instructions. Each column was prepared by adding 600 ml of binding buffer and allowing it to pass the resin bed by gravity flow. Then, 60 ml of plasma were diluted in 540 ml of binding buffer applied to the affinity column, to accomplish the specific binding of albumin and IgG. The eluate was collected to 1200 ml of binding buffer, used to wash the column.

### Two-dimensional Gel Electrophoresis

The first-dimensional gel separation was carried out with ReadyStrip™ IPG strips (Bio-Rad, CA, USA) following the manufacturer's protocol. About 500 µg protein of plasma for gel were diluted to 170 µl with re-hydration solution (5 M urea, 2M Thiourea, 2% CHAPS, 100 mM DTT, 0.5% v/v pH 3–10 IPG buffer, 40 mM Tris Base, 2% SB 3–10, and trace bromophenol blue), and applied to immobilized 17 cm pH 3–10 nonlinear gradient strips by over-night re-hydration at 50 V. Isoelectric focusing (IEF) were performed with an Ettan IPGphor II apparatus (GE Healthcare) as follow steps: 0–500 V (500 Vh), 500 V (2500 Vh), 500–3,500 V (10,000 Vh), 3,500 V (50,000 Vh), 3,500–500 V (8000 Vh) for a total of 7.1 kVh. All IEF steps were carried out at 20°C. After the first-dimensional IEF, IPG gel strips were placed in an equilibration solution (6 M urea, 2% SDS, 30% glycerol, 50 mM Tris-HCl, pH 8.8) containing 1% DTT for 10 min with shaking at 50 rpm on an orbital shaker. The gels were then transferred to the equilibration solution containing 2.5% iodoacetamide and shaken for a further 10 min before placing them on 12% polyacrylamide gel slab.

Separation in the second dimension was carried out by using Protean II electrophoresis equipment and Tris-glycine buffer (25 mM Tris, 192 mM glycine) containing 0.1% SDS, at a current setting of 5 mA/gel for the initial 1 h, and 10 mA/gel thereafter. The second-dimensional SDS-PAGE was developed until the bromophenol blue dye marker had reached the bottom of the gel. This process was repeated in each pooling sample for three times.

### Image Analysis

After 2-DE separation, the nine gels were stained with Coomassie blue R-250 (Sigma, St Louis, MO, USA). Spot detection and quantification were carried out using PDQuest 2D-analysis software (Bio-Rad, Hercules, CA). Spot intensity was quantified automatically by calculation of spot volume after normalization of the image by taking the ratio of intensity of one spot to the total spots, and expressed as a fractional intensity. Only those spots with significant change (un-paired *t*-test, *P*<0.01) in expression intensity were selected for mass spectrometry analysis.

### Tryptic Digestion

Protein spots were excised from the gel with the Ettan Spot Picker and destained with 25 mM ammonium bicarbonate, 50% acetonitrile. Gels were then dried completely by centrifugal lyophilization. Each spot was digested overnight in 8 µ 10.1 mg/ml trypsin for 16 h at 37°C. The peptides were extracted three times with 50% acetonitrile, 0.1% trifluoroacetic acid, and dried completely by centrifugal lyophilization.

### MALDI/TOF MS

Before the analysis, external calibration with angiotensin II and ACTH 18–39 was employed. This procedure typically results in mass accuracies of 100 ppm or better. Then, the peptide mixture (0.3 µl) was mixed with α-cyano-4-hydroxycinnamic acid matrix (1∶1, v/v) and analyzed on a MALDI/TOF MS (Autoflex, Bruker Daltonics, Germany), operated in the delayed extraction and reflector mode. The laser wavelength was 337 nm, and the laser repetition rate was 3 Hz. The MALDI spectra were averaged over 200–400 laser shots. MALDI generally produces the protonated molecule ion.

### Database Searches for Protein Identification

These mass spectra were interpreted with GPS Explorer software (Applied Biosystems, Foster City, CA,) using the MASCOT search engine (http://www/matrixscience.com) for protein identification by peptide mass finger-printing (PMF). Search parameters were set as follows: precursor tolerance: ±0.15 Da; missed cleavages: 1; fixed modifications: carbamidomethyl; variable modifications: oxidation. The MASCOT score from the sample with ammonium phosphate was higher than that from the sample without ammonium phosphate, thus increasing the confidence in the correct identification of the proteins.

### Enzyme-Linked Immunosorbent Assay

Human enzyme-linked immunosorbent assay (ELISA) kits (Uscn, Life Science Inc. USA) were used to detect candidate proteins level. The methods followed the manufacturer's instructions. Briefly, 100 µl of a Standard was dispensed into each of ten wells, and 100 µl of specimens were dispensed into other plate wells. After dispensing 50 µl of enzyme conjugate reagent into each well, the solutions were gently mixed for 15 s. The plate was then incubated at 37°C for 30 min. After removal of the mixture from the incubator, the microtiter wells were rinsed with deionized water and emptied five times. The wells were sharply stricken onto absorbent paper to remove residual water droplets. Subsequently, 50 µl of color A and color B reagent was respectively added to each well, and the solutions were incubated at 37°C for 15 min. The reaction was stopped with the addition of 50 µl of stop solution into each well and gently mixed for 30 s (the blue color changes completely to yellow). Optical density is read at 450 nm within 15 min in a microtiter plate reader.

### Statistical Analysis

The Statistical Package for the SPSS 10.0 (SPSS Inc., Chicago, USA) and GraphPad Prism 5 Demo (GraphPad Software, San Diego, California, USA) were used for statistical analysis. Data were expressed as mean ± SEM. Unpaired *t*-test was used to compare values between control and VHD groups. Statistical significance was defined as *P*<0.05.

## Results

### 2-DE Analysis and SELDI/TOF MS Identification

**Figure 1:**
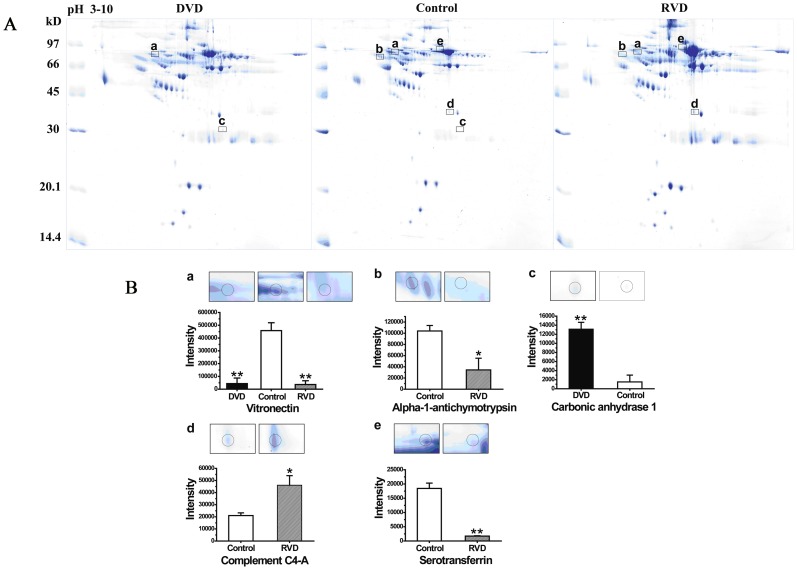
Representative two-dimensional gel (2-DE) of plasma in valvular heart disease and normal controls. (A) 500 µg protein of plasma were subjected to 2-DE with first dimension pH gradient of 3–10 and second dimension SDS-PAGE. Gels were stained with colloidal Coomassie Blue R-250. Spots were excised, processed as described under “Experimental Procedures”, and analyzed by peptide mass fingerprinting. (B) a. The intensity of plasma vitronectin in RVD (3622.9±2975.6) and DVD (4351.4±4351.4) was shown down-regulate in expression to compare with the normal controls groups (45793.3±6190.2). b. The intensity of plasma alpha-1-antichymotrypsin in RVD (34446.9±20890.9) was shown down-regulate in expression to compare with the normal controls groups (104007.5±9878.797). c. The intensity of plasma carbonic anhydrase 1 in DVD (13153.3±1485.5) was shown up-regulate in expression to compare with the normal controls groups (1502.6±1502.6). d. The intensity of plasma complement C4-A in RVD (46090.8±7914.7) was shown up-regulate in expression to compare with the normal controls groups (21009.9±2250.4). e. The intensity of plasma serotransferrin in RVD (1712.6±112.7) was shown down-regulate in expression to compare with the normal controls groups (18444.9±1861.3) Date are shown as mean ± SEM. (n = 3, *P<0.05 vs. control group, **P<0.01 vs. control group, un-paired *t*-test).

In the pilot study, plasma from 20 patients with RVD and 20 patients with DVD were studied with comparison to the plasma from 20 normal controls (Figure 1A). Samples were processed by immune-affinity depletion of the albumin, and IgG. Differential protein analysis was then performed by using 2-DE, as previously described. A total of 473 protein spots were detected in all three groups. It was found that there were 11 protein spots significantly down-regulated (*P*<0.05 versus control, unpaired *t*-test) and 13 protein spots significantly up-regulated (*P*<0.05 versus control, unpaired *t*-test) in the plasma of patients.

All 24 protein spots of interest were trypsin-digested and MALDI-TOF MS was used for the identification of the protein spots. The results indicated that 18 protein spots and the 14 corresponding proteins or polypeptides could be identified (Table 2). In contrast, 6 protein spots did not have PMF or did not have corresponding proteins in the MASCOT database.

### Significantly altered protein expression in patients with RVD and DVD

In eight down-regulated proteins, six proteins or polypeptides were identified in RVD group, three proteins or polypeptides were identified in DVD group, and one corresponding protein or polypeptide was identified in both two comparative groups (Table 4). In six up-regulated proteins, three proteins or polypeptides were found in RVD group, five proteins or polypeptides were found in DVD group, and two proteins or polypeptides were identified in both two comparative groups (Table 4).

**Table 4:** Differentially Expressed Proteins Identified by MALDI/TOF MS.

Protein identification	Groups	MW(Da)	pI	Accession No.	Protein Score	Protein Score CI %	Main Protein Function #
**Protein spots down-regulated in the plasma of patients**							
Alpha-1-antichymotrypsin *	RVD	47791.6	5.33	P01011	142.0	100.0	Inhibit neutrophil cathepsin G and mast cell chymase, both of which can convert angiotensin-1 to the active angiotensin-2.
Elongation factor 2	RVD	81909.1	7.19	Q9YC19	78.1	99.2	Catalyzes the GTP-dependent ribosomal translocation step during translation elongation.
Alpha-1B-glycoprotein	RVD	54789.8	5.56	P04217	81.3	99.6	Unclear.
Fibrinogen gamma chain	DVD	52106.1	5.37	P02679	129.0	100.0	Fibrinogen has a double function: yielding monomers that polymerize into fibrin and acting as a cofactor in platelet aggregation.
Hemopexin	DVD	52322.6	5.44	Q5R543	74.9	98.3	Binds heme and transports it to the liver for breakdown and iron recovery, after which the free hemopexin returns to the circulation
Serotransferrin *	RVD	79294.5	6.81	P02787	274.0	100.0	Responsible for the transport of iron from sites of absorption and heme degradation to those of storage and utilization.
Fibrinogen alpha chain	RVD	95655.6	5.70	P02671	209.0	100.0	Fibrinogen has a double function: yielding monomers that polymerize into fibrin and acting as a cofactor in platelet aggregation.
Fibrinogen alpha chain	RVD	95655.6	5.70	P02671	226.0	100.0	
Vitronectin *	RVD & DVD	55069.5	5.55	P04004	126.8	100.0	Serves as a cell-to-substrate adhesion molecule; Inhibitor of the membrane-damaging effect of the terminal cytolytic complement pathway.
**Protein spots up-regulated in the plasma of patients**							
Carbonic anhydrase 1 *	DVD	28909.4	6.59	P00915	98.5	100.0	Reversible hydration of carbon dioxide; Hydrates cyanamide to urea.
Complement C4-A *	RVD	194247.1	6.65	P0C0L4	97.9	100.0	C4 plays a central role in the activation of the classical pathway of the complement system. C4-A anaphylatoxin is a mediator of local inflammatory process. It induces the contraction of smooth muscle, increases vascular permeability and causes histamine release from mast cells and basophilic leukocytes.
Zinc-alpha-2-glycoprotein	RVD & DVD	34465.2	5.71	P25311	91.9	100.0	Stimulates lipid degradation in adipocytes and causes the extensive fat losses associated with some advanced cancers.
Haptoglobin	DVD	45860.8	6.13	P00738	77.5	99.1	Haptoglobin combines with free plasma hemoglobin, preventing loss of iron through the kidneys and protecting the kidneys from damage by hemoglobin, while making the hemoglobin accessible to degradative enzymes.
Haptoglobin	DVD	45860.8	6.13	P00738	139.0	100.0	
Haptoglobin	DVD	45860.8	6.13	P00738	82.2	99.7	
Ig kappa chain C region	DVD	11772.7	5.58	P01834	99.8	100.0	Defects in IGKC are the cause of immunoglobulin kappa light chain deficiency (IGKCD).
Ig alpha-1 chain C region	RVD	38485.9	6.08	P01876	76.7	98.9	Ig alpha is the major immunoglobulin class in body secretions. It may serve both to defend against local infection and to prevent access of foreign antigens to the general immunologic system.
Ig alpha-1 chain C region	RVD & DVD	38485.9	6.08	P01876	112.0	100.0	

RVD = rheumatic valvular disease; DVD = degenerative valvular disease; MW = protein molecular weight; pI = isoelectric point.

* Candidate proteins for validation;

# UniProt Knowledgebase, http://expasy.org/uniprot.

We subsequently focused on several proteins that may be the surrogate biomarkers of disease processes. Three down-regulated proteins (Alpha-1-antichymotrypsin, Serotransferrin, and Vitronectin) and two up-regulated proteins (Complement C4-A, and Carbonic anhydrase 1) were chosen to be candidate proteins for validation (Figure 1 B).

### Validation of the Candidate Protein by ELISA

**Figure 2:**
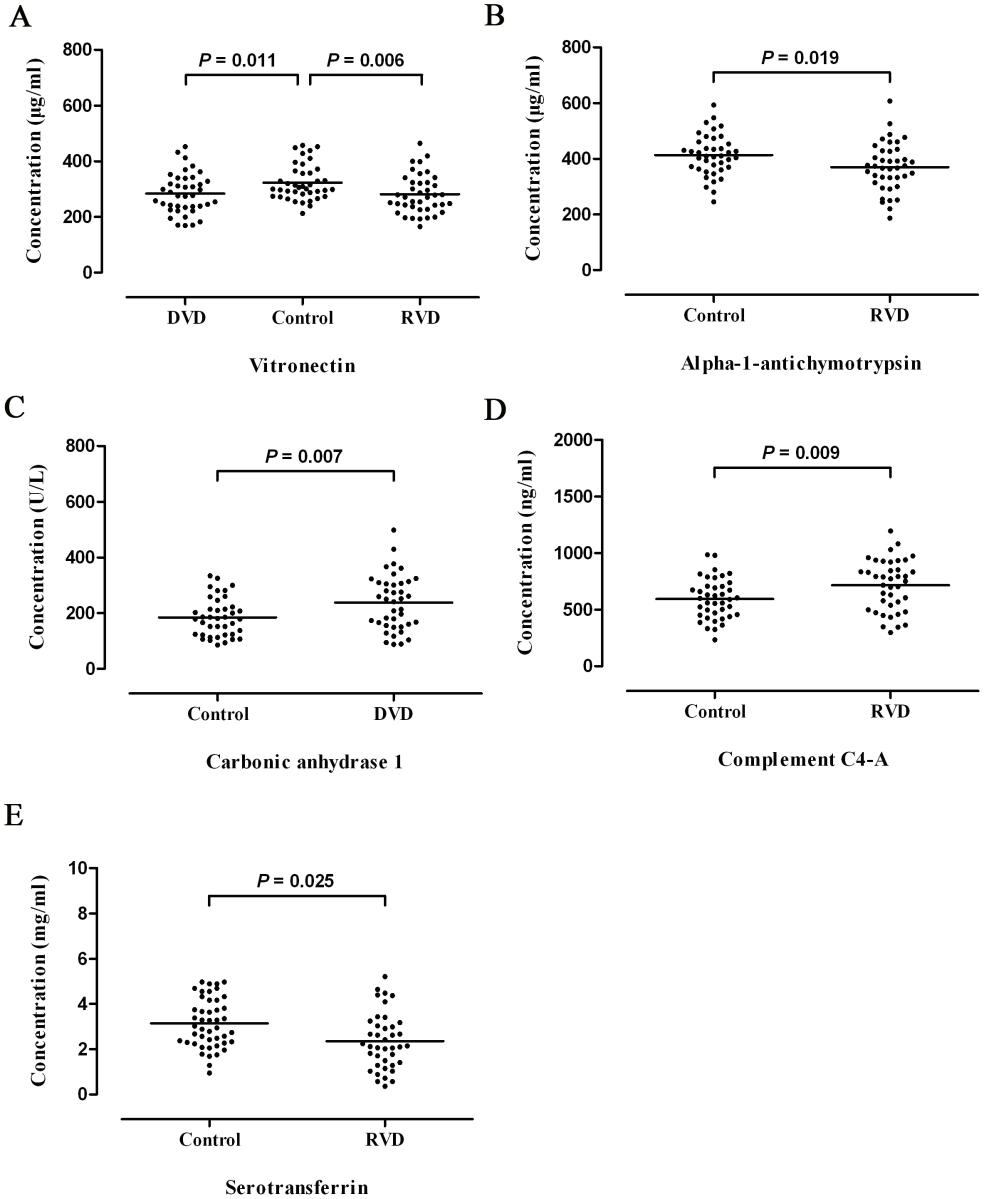
ELISA was used to measure plasma levels of vitronectin, alpha-1-antichymotrypsin, carbonic anhydrase 1, complement C4-A and serotransferrin. (A) The plasma vitronectin level in normal controls (323.2±10.0 µg/ml) were significantly elevated when compared with the patients with RVD (281.3±11.0 µg/ml, *P* = 0.006; un-paired *t*-test) and patients with DVD (283.6±11.4 µg/ml, *P* = 0.011; un-paired *t*-test). (B) Plasma alpha-1-antichymotrypsin levels in patients with RVD (370.0±13.7 µg/ml) were significantly depressed when compared with the healthy controls (413.0±11.6 µg/ml, *P* = 0.019; un-paired *t*-test). (C) Plasma carbonic anhydrase 1 levels in patients with DVD (237.70±15.7 U/L) were significantly elevated when compared with the healthy controls (184.7±10.8 U/L, *P* = 0.007; un-paired *t*-test). (D) Plasma complement C4A levels in patients with RVD (715.8±35.6 ng/ml) were significantly elevated when compared with the healthy controls (594.7±28.2 ng/ml, *P* = 0.009; un-paired *t*-test). (E) Plasma serotransferrin levels in patients with RVD (2.36±0.20 mg/ml) were significantly depressed when compared with the healthy controls (2.93±0.16 mg/ml, *P* = 0.025; un-paired *t*-test). Date are shown as mean ± SEM. (n = 40).

Plasma concentration in patients with RVD (n = 40), DVD (n = 40), and normal controls (n = 40) were measured by ELISA. The plasma vitronectin level in patients with RVD was significantly lower than those in normal controls (281.3±11.0 vs. 323.2±10.0 µg/ml, *P* = 0.006; un-paired *t*-test; Figure 2A). The same alternation was also detected in plasma of patients with DVD (283.6±11.4 vs. 323.2±10.0 µg/ml, *P* = 0.011; un-paired *t*-test; Figure 2A).

The plasma alpha-1-antichymotrypsin level in RVD patients was significantly lower than those in normal controls (370.0±13.7 vs. 413.0±11.6 µg/ml, *P* = 0.019; un-paired *t*-test; Figure 2B).

In addition, the plasma carbonic anhydrase 1 level in DVD patients was significantly higher than those in normal controls (237.70±15.7 vs. 184.7±10.8 U/L, *P* = 0.007; un-paired *t*-test; Figure 2C).

The plasma complement C4A level in RVD patients was significantly higher than those in normal controls (715.8±35.6 vs. 594.7±28.2 ng/ml, *P* = 0.009; un-paired *t*-test; Figure 2D).

Finally, the plasma serotransferrin level in RVD patients was significantly lower than those in normal controls (2.36±0.20 vs. 2.93±0.16 mg/ml, *P* = 0.025; un-paired *t*-test; Figure 2E).

## Discussion

We have analyzed the changes in different proteins expressed in the plasma of VHD patients including RVD and DVD by using proteomic analysis. To our knowledge, this is the first proteomic study on the altered plasma proteins in various VHD.

In this study, we have, for the first time, found that 1) alterations of 14 differential proteins or polypeptides by using the 2-DE and MALDI/TOF MS in the plasma of patients with VHD including RVD and DVD; 2) the elevation of plasma complement C4A in RVD and carbonic anhydrase 1 in DVD and the decrease of serotransferrin and alpha-1-antichymotrypsin in RVD patients may be useful biomarkers for these valvular diseases; and 3) the decreased plasma level of vitronectin – a protein related to the formation of valvular structure – in both RVD and DVD patients might indicate the possible genetic deficiency in these patients.

In the present study, we compared the plasma protein expression alterations between VHD patients (RVD or DVD) and normal controls by using 2-DE combined with MALDI/TOF MS approach. A total of 14 differentially expressed proteins were identified in the plasma of VHD patients. In the plasma of RVD patients, six proteins or polypeptides were down-regulated (alpha-1-antichymotrypsin, elongation factor 2, alpha-1B-glycoprotein, serotransferrin, fibrinogen alpha chain, vitronectin) and three (complement C4-A, Ig alpha-1 chain C region, zinc-alpha-2-glycoprotein) were up-regulated. Moreover, three proteins or polypeptides (hemopexin, fibrinogen gamma chain, vitronectin) were down-regulated and five (carbonic anhydrase 1, zinc-alpha-2-glycoprotein, haptoglobin, Ig kappa chain C region, Ig alpha-1 chain C region) were up-regulated in the plasma of DVD patients.

The genetic basis and proteomics of common VHD are important to provide information for diagnosis and treatment of these diseases. In fact, the proteome reflects all proteins and peptides that may be related to certain genes and proteomics allows a more detailed evaluation of disease status. In particular, plasma is the ideal source for proteome analysis as previously discussed [16]. The molecular events into VHD progression are complex and diverse, and they remain incompletely characterized. The identification, quantification, classification, and functional assignment of proteins are essential to the full understanding of these molecular events. Such information may likely prove to be crucial in disease process cognition, pathological changes exploration, therapeutic targets discovery, rational drugs design and may ultimately affect patient outcomes [17].

2-DE is currently a widely used analytical method for proteomic studies. It allows the separation of a mixture of proteins by intrinsic charges in the first dimension and by relative molecular masses in the second dimension. The characteristic 2-DE migration pattern of each individual protein offers a powerful tool for protein separation and identification [18]. Developments in technology and instrumentation have made mass spectrometry the method of choice for the identification of gel-separated proteins using rapidly growing sequence databases [19]. Proteins with a full-length sequence present in a database can be identified with high certainty and high throughput by using the accurate masses obtained by MALDI/TOF MS peptide mapping. Therefore, 2-DE combined with MALDI/TOF MS has become a useful approach for proteomics studies [20]–.

From the present study, we used ELISA to validate the findings in the 2-DE combined with MALDI/TOF MS in enlarging samples. Several proteins that may be the surrogate biomarkers of disease processes as candidate proteins were validated. The results confirmed that the mean plasma level of these candidate proteins in VHD patients was significantly altered compared with normal controls.

Carbonic anhydrase 1 is the first member of carbonic anhydrases (CAs) family. CAs are zinc metalloenzymes that catalyze the reversible hydration-dehydration of carbon dioxide and bicarbonate [24]. At least 11 members of the CA family identified at present, with CA2 being the most abundant and most efficient enzyme. CAs appear to have a role in diverse physiological and biological processes including calcification, acid-base balance, ion transport, and bone absorption [24]–[27]. *In vitro* assays demonstrated that carbonic anhydrase 1 not only enhances the hydration reaction but also promotes the formation of CaCO_3_ [28]–[30]. Calcium salt precipitation is an important step in tissue calcification. Thus, the increased carbonic anhydrase 1 expression may lead to improper mineralization by accelerating calcium salt deposition [31]. Moreover, valvular calcification is one of the common and key pathological changes in DVD. In the present study, we found that carbonic anhydrase 1 was up-regulation in the plasma of DVD patients by using two different methods of 2-DE-MALDI/TOF MS and ELISA. Obviously, the up-regulation of carbonic anhydrase 1 might lead to valve calcification by accelerating calcium salt deposition.

Complement C4-A is one isotype of Complement C4. Complement C4 is an essential component of the effector arm of the humoral immune response. It plays a central role in the activation of the classical pathway of the complement system. Complement C4 positions at the pivotal point by which the activation of the classical pathway and the lectin pathway is accomplished [32]. Downstream of C4 activation includes the activation of C3 and C5, the generation of the anaphylatoxins, the initiation of the lytic pathway, the opsonization and immune clearance processes, and the communication with other branches of the immune system to achieve immune tolerance and to potentiate the humoral immune response [33], [34]. C4 is the most polymorphic component of the complement system. While examining the strength of the host defense or the susceptibility of an individual to microbial infections, it is desirable to include the status of C4A and C4B into consideration [32]. RVD is also the result of valvular damage caused by an abnormal immune response to group A streptococcal infection [35]. Moreover, many investigators have examined the relationship between phenotypic absence or partial deficiencies of complement C4A in autoimmune diseases such as systemic lupus erythematosus and autoimmune hepatitis [36]–[39]. Man XY and associates found that deficiency of C4A, but not C4B or C2, may be a risk factor for acquiring SLE in south west Han Chinese [37]. Scully LJ and associates also reported that a C4A gene deletion is found in patients with autoimmune hepatitis, especially those presenting at a young age. This complement gene deletion may be an important factor in the development of this disease [38]. Importantly, it was reported that the rare C4A*6 allele was significantly increased in the RVD patients, suggesting that C4A allele might be related to RVD [39]. In our study, we found that complement C4-A was up-regulated in the plasma of RVD patients, suggesting this protein might be related to pathology of RVD.

Alpha-1-antichymotrypsin, a member of the serine proteinase inhibitor family, inhibits neutrophil proteinase cathepsin G and mast cell chymases, and protects the lower respiratory tract from damage by proteolytic enzymes. It contains a reactive centre loop, which interacts with cognate proteinases, resulting in loop cleavage and a major conformational change. Alpha-1-antichymotrypsin has been implicated in the pathology of a number of devastating human diseases including chronic obstructive pulmonary disease (COPD), Parkinson's disease (PD), Alzheimer's disease (AD), stroke, cystic fibrosis, cerebral haemorrhage and multiple system atrophy [40]. Chopra P and associates [41] affirmed the genesis of Aschoff bodies by using alpha-1-antichymotrypsin as histiocytic marker. Aschoff bodies are nodules found in the hearts of individuals with rheumatic fever. They result from inflammation in the heart muscle and are characteristic of RVD. In the present study, alpha-1-antichymotrypsin was down-regulated in the plasma of RVD patients. However, the role of this protein in the pathology of RVD remains unclear.

Serotransferrin is an iron binding transport protein that can bind two Fe^3+^ ions in association with the binding of an anion, usually bicarbonate. It is responsible for the transport of iron from sites of absorption and heme degradation to those of storage and utilization. Serotransferrin plays a role in the stimulation of cell proliferation; it can also act as an insulin antagonist, producing acute hyperglycemia in normoglycemic rats and ketonuria in diabetic rats. Further, this protein is also involved in tumoral processes, correlating with severity of disease and promoting endothelial cell migration and invasion [42]. Among its multiple biological functions, an important antibacterial activity is impressive [42]–[45]. As mentioned above, RVD is the result of valvular damage caused by an abnormal immune response to group A streptococcal infection [35]. In the present study, the plasma serotransferrin level in RVD patients was significantly lower than those in normal controls. It is possible that low level of serotransferrin might be susceptible to group A streptococcal infection.

Vitronectin belongs to the group of Arg-Gly-Asp (RGD)-type adhesive glycoproteins that play key roles in the attachment of cells to their surrounding extracellular matrix (ECM) and participate in the regulation of cell migration/invasion, proliferation, and tissue remodeling. As a “matricellular” protein, vitronectin does not subserve a primary role in structuring the ECM but rather acts as modulator of the cell-ECM interface, particularly apparent in the vascular system and in association with tumors. Vitronectin exerts regulatory functions in the control of hemostasis, blood coagulation, and pericellular proteolysis as well as in innate immunity, especially related to complement regulation including complement C4A, leukocyte recruitment, or bacterial tropism [46]. In fact, complement C4A is up-regulated in RVD patients in the present study as mentioned above. Coordination of cell adhesion/invasion and pericellular proteolysis by vitronectin is mediated by specific binding interactions with cell surface receptors (such as αv integrins and the urokinase receptor) as well as with humoral proteins (particularly proteases and their inhibitors). For example, binding of plasminogen activator inhibitor (PAI)-1 to vitronectin not only results in stabilization of the serine protease inhibitor at sites of cell migration and invasion but also contributes to proteolysis-independent antiadhesive functions of PAI-1 that are determined by its “cofactor” vitronectin [47]. As discussed above, alpha-1-antichymotrypsin, a member of serine protease inhibitor family, is down-regulated in DVD patients and this may suggest a possible correlation between vitronectin and alpha-1-antichymotrypsin in the pathology of valvular disease.

Moreover, the detailed structural analysis of specific binding sites in vitronectin has been instructive to further clarify the role of the multitalented adhesive protein in thrombosis and hemostasis or in different vascular pathologies. Akhtar and colleagues [48] identified vitronectin in both normal and myxomatous mitral heart valve and found the amount of this protein increased in the diseased tissue. In addition, Bouchey and colleagues [49] found that vitronectin played a role in avian cardiac valve development. Based on this, the finding from the present study that vitronectin in the plasma was down-regulation in both RVD and DVD patients may suggest that alteration of this protein might be related to valvular pathological changes.

In addition, to what extend the differences found in the present study between RVD and DVD are related to the consequence of the VHD, such as left ventricular hypertrophy, is unclear. This warrants future investigations.

There are some other differentially expressed proteins found in our study (shown in Table 4). However, it remains to define the role of these proteins in the pathology of VHD and the clinical significance in VHD.

### Limitation of Study

Some limitations of this study should be discussed as follows. (1) Although 2-DE is now widely used as the solution to detect differential protein expression, some problems of this technique should not be ignored. First, the loss of proteins in the 2-DE analysis remains a serious limitation to the concept of 2-DE as a global approach at present, specifically hydrophobic as well as large proteins above 100 kDa. Second, proteins whose pI values fall at the extremities of the pH gradients are difficult to resolve on a gel. In addition, reproducibility is also a limitation with 2-DE. To eliminate this limitation maximally, in our study, in order to enhance the experimental repeatability, every pooling plasma groups were performed 2-DE three times, and statistical method combine with standard (*P*<0.05) were used to evaluate the difference of protein spots. (2) A limitation of MALDI/TOF MS is the identification of low molecular mass proteins. In general, identification of small weight proteins (<500 Da) by MALDI/TOF MS is not efficient. Currently, no single protein database is sufficient in characterizing all useful PMF spectra generated by the MALDI/TOF MS instrument. In the present study, six protein spots were not identified because of the above limitations.

In summary, in this study, we have, for the first time, identified alterations of 14 differential proteins or polypeptides by using the 2-DE and MALDI/TOF MS in the plasma of patients with VHD including RVD and DVD. Moreover, the elevation of plasma complement C4A in RVD and carbonic anhydrase 1 in DVD and the decrease of serotransferrin and alpha-1-antichymotrypsin in RVD patients may be useful biomarkers for these valvular diseases. In addition, the decreased plasma level of vitronectin – a protein related to the formation of valvular structure – in both RVD and DVD patients might indicate the possible genetic deficiency in these patients.
